# 

**Published:** 1891-06

**Authors:** 


CONTENTS:

PAGE

ORIGINAL ARTICLES:
- The Oxwarble of the United States. By Cooper Curtice, Vet. ...... 265
- The Prevalence of Tuberculosis. By Daniel D. Lee, M. D. V. ...... 274
- Nail Wound of the Foot. By A. W. Clement, V. S. ...... 277
- Thrombosis in Iliac Arteries. By J. C. Huilson, V. S. ...... 279
- Age of the Horse, Ox, Dog, and Other Domesticated Animals. By R. S. Huidekoper, M. D., Vet. ...... 282
- Recent Patents. ...... 286

EDITORIAL:
- Veterinary Legislation ...... 290
- 2nd Congress for Study of Tuberculosis ...... 294
- Indian Journal ...... 294

SELECTIONS FROM FOREIGN JOURNALS:
- German Review ...... 295
- Action of Koch's Lymph in Bovine Tuberculosis ...... 298
- Sheep Dips ...... 301
- Tuberculosis in Monkey ...... 304

SOCIETY PROCEEDINGS:
- Keystone Veterinary Medical Association ...... 305
- Massachusetts Veterinary Association ...... 306

COMMUNICATIONS:

- Osteo Porosis. By A. Jasme, V. S ...... 307

NECROLOGY.

- Joseph Leidy ...... 308

- Leon Lafosse ...... 310

- Nostrand ...... 310

REVIEW DEPARTMENT:
- Veterinary Surgery ...... 311
- Books and Pamphlets Received ...... 312

